# Evaluation of antioxidant and cytotoxic properties of phenolic *N*-acylhydrazones: structure–activity relationship

**DOI:** 10.1098/rsos.211853

**Published:** 2022-06-08

**Authors:** Jovica Branković, Nevena Milivojević, Vesna Milovanović, Dušica Simijonović, Zorica D. Petrović, Zoran Marković, Dragana S. Šeklić, Marko N. Živanović, Milena D. Vukić, Vladimir P. Petrović

**Affiliations:** ^1^ University of Kragujevac, Faculty of Science, Department of Chemistry, R. Domanovića 12, 34000 Kragujevac, Serbia; ^2^ University of Kragujevac, Institute for Information Technologies, Kragujevac, Department of Science, Jovana Cvijića bb, 34000 Kragujevac, Serbia; ^3^ University of Kragujevac, Faculty of Agronomy in Čačak, Ljubićska 30, Čačak, Serbia

**Keywords:** phenolic *N*-acylhydrazones, antioxidant activity, cytotoxic activity, structure–activity relationship, density functional theory

## Abstract

Cancer is still a relentless public health issue. Particularly, colorectal cancer is the third most prevalent cancer in men and the second in women. Moreover, cancer development and growth are associated with various cell disorders, such as oxidative stress and inflammation. The quest for efficient therapeutics is a challenging task, especially when it comes to achieving both cytotoxicity and selectivity. Herein, five series of phenolic *N*-acylhydrazones were synthesized and evaluated for their antioxidant potency, as well as their influence on HCT-116 and MRC-5 cells viability. Among 40 examined analogues, 20 of them expressed antioxidant activity against the DPPH radical. Furthermore, density functional theory was employed to estimate the antioxidant potency of the selected analogues from the thermodynamical aspect, as well as the preferable free-radical scavenging pathway. Cytotoxicity assay exposed enhanced selectivity of a number of analogues toward cancer cells. The structure–activity analysis revealed the impact of the type and position of the functional groups on both cell viability and selectivity toward cancer cells.

## 1. Introduction

**Figure 1.**
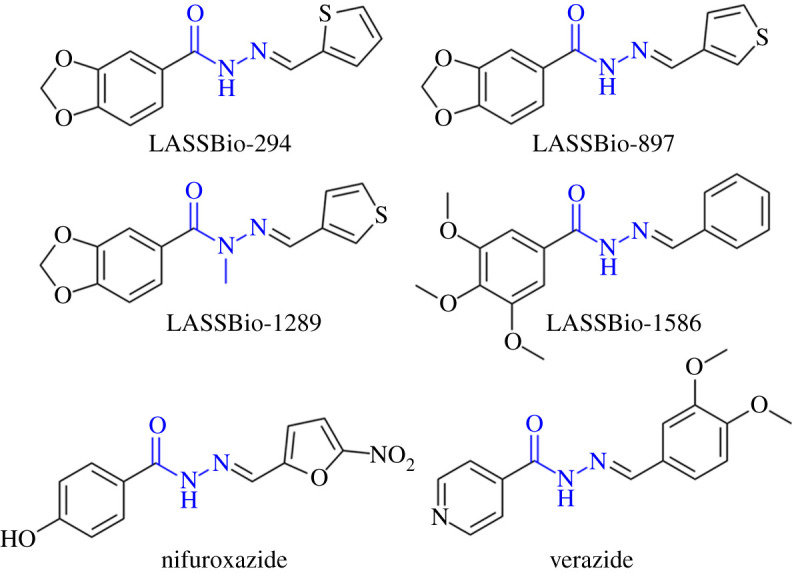
***N*AH**-based drugs.

The multi-target concept in drug discovery has made rapid advancements since its introduction at the beginning of the twenty-first century [1]. Regardless of increased interest in multi-target drugs, the 'one-molecule, one-target, one-disease' approach is still a principally employed strategy [2]. The treatment of complex diseases, such as Alzheimer's, Parkinson's, and cancer, is suggested to be more beneficial by employing drugs targeting multiple aetiologies of the same disease [3]. Such multifunctional drugs exhibiting multiple mechanisms of action are considered to possess fewer side effects in comparison to several drug combination strategies [3]. Despite the ongoing efforts in drug design and discovery, cancer is still an emerging worldwide problem, and the search for efficient therapeutics seems to be a never-ending task. Moreover, cancer initiation and progression are linked with various cell disorders, such as oxidative stress and chronic inflammation [4]. Following the multifunctional approach, the bioactive compounds with multiple synergized activities could be one of the pathways to the solution of the cancer problem. In this respect, one can take advantage of the valuable chemotherapeutic potential of hydrazone-type compounds [5]. Hydrazones correspond to a variety of naturally occurring and synthetic organic compounds characterized by the N–N bond integrated within the R^1^R^2^C = N–NR^3^R^4^ skeleton [6]. This pharmacophore is appointed as a promising structural motif in the field of medicinal chemistry, due to the diverse biological and pharmacological properties of hydrazone-type compounds [5,7]. Hydrazone-type derivatives are known for their antioxidant [8–13], antitumoral [14–19], anti-inflammatory [20–22], antimicrobial [23–25], anti-Parkinson [26], anti-Alzheimer [13], antimalarial [27], antidiabetic [28,29], antiatherogenic [8,9], antifungal [30], antibacterial [31], antiplatelet [21], antiviral [32,33] and many other activities. Hydrazone nucleus, as a fusion of amide and imine subunits, possess hydrogen-bond donor and acceptor sites for interaction with amino acid residues [18]. Thus, compounds with hydrazone cores express inhibitory effects against numerous enzymes, such as cyclooxygenase (COX-1 and COX-2) [20,34], acetyl- and butyrylcholinesterase (AChE and BuChE) [35], monoamine oxidase A (MAO A) [36], as well as G-Protein-Coupled Receptor Kinase 2 (GRK2) involved in heart failure [37]. Particularly, the *N*-acylhydrazone group (***N*AH**) is declared as a unique and versatile structural motif, suitable for synthetic transformations and the development of potential therapeutically useful compounds [38]. Compounds containing the ***N*AH** group are well-known for their anticancer and anti-inflammatory properties [39,40]. ***N*AH** core is useful for the synthesis of small-molecule scaffolds which is especially attractive for medicinal chemists [38]. Some of the FDA-approved drugs bearing the ***N*AH** motif are antibiotic nifuroxazide and tuberculostatic verazide (figure 1) [24,38,41]. Although hydrazone derivatives were investigated for years, the encouragement of further studies on hydrazone-type compounds is still active [6]. A number of potential anti-cancer drugs containing ***N*AH** backbone are in the phase of a preclinical or clinical trial [19]. Hydrazones can also be used as drug carriers and for the controlled release of anti-cancer drugs in tumour sites [42].

The versatility of hydrazone-type compounds inspired us to use the ***N*AH** scaffold as a framework for the synthesis of phenolic ***N*AH** derivatives (**Phe*N*AH**s**)** and to investigate their antioxidant and cytotoxic properties. According to the statistical data acquired from the American Institute for Cancer Research, colorectal cancer is the third most commonly occurring cancer in men and the second most commonly occurring cancer in women.

Such alarming numbers prompted us to assess the cytotoxic activity of synthesized phenolic *N*-acylhydrazones on HCT-116 and MRC-5 cell lines. Furthermore, the relationship between carcinogenesis, inflammation and reduction/oxidation cell disbalance motivated us to examine their potential dual antioxidant/cytotoxic nature *in vitro*. All these encouraged us to perform structure–activity analysis to investigate the influence of different substituents on antioxidant and cytotoxic activities.

## 2. Material and methods

All chemicals were acquired either from Sigma-Aldrich Co. or Merck & Co. Benzohydrazides (**1**–**5**) were prepared according to the previously reported method [43]. The IR spectra were recorded on a PerkinElmer Spectrum One FT-IR spectrometer using the KBr plates. The UV-Vis spectra were measured within the 200–500 nm range on the Agilent Technologies, Cary 300 Series UV-Vis Spectrophotometer. The ^1^H NMR and ^13^C NMR spectra were run on a Varian Gemini spectrometer (200 MHz for ^1^H and 50 MHz for ^13^C) using dimethyl sulfoxide-d6 (DMSO-d6) as solvent. A Shimadzu Prominence High Performance Liquid Chromatography (HPLC) system consisting of LC-20AT pump, DGU-20A degasser, CTO-20A column oven, 20 µl loop, an A Luna C18 column (250 × 4.6 mm, 5 µm, Phenomenex, USA), SPD-M20A PDA detector (at 254 nm) and CBM-20 A Prominence communication module was employed to determine the purity of compounds. The mobile phase consisted of (A) acetonitrile and (B) water. The following gradient program was used: 0–5 min, 50% A and 50% B; 5–10 min, 60% A and 40% B. The column oven was adjusted at 35°C and the flow rate was 1 ml min^−1^.

### 2.1. General procedure for the synthesis of phenolic *N*-acylhydrazone (Phe*N*AH) derivatives

A mixture of equimolar amounts of the corresponding benzohydrazide (1 mmol) and aldehyde (1 mmol) in ethanol as a solvent (3 ml) was heated to 80°C for 3 h. Reaction progress was monitored by thin-layer chromatography (TLC). After the completion of the reaction, the formed precipitation was filtrated and washed with water. All products were characterized by ^1^H NMR, ^13^C NMR, UV-Vis and FT-IR spectra, whereas the purity was determined by HPLC. The spectral characterization and corresponding spectra for all synthesized compounds are given in electronic supplementary material.

### 2.2. Determination of the antioxidant activity of Phe*N*AHs

The antioxidant screening of all **Phe*N*AH**s was performed using the 2,2-diphenyl-1-picrylhydrazyl (DPPH) method [44]. The solution of DPPH radical in methanol (0.05 mM, 1 ml) was mixed with the tested compound (20 µl of different concentrations in dimethyl sulfoxide (DMSO) and 980 µl of methanol). The reaction mixture was incubated in the dark at room temperature for 20 and 60 min after which the absorbance of the sample was measured spectrophotometrically at 517 nm. All measurements were performed in triplicate. Methanol was used as a control solution, whereas quercetin and nordihydroguaiaretic acid (NDGA) were used as reference compounds. IC_50_ values were determined for all compounds which exhibited good activity. IC_50_ is defined as the minimal concentration of tested compound required for reaching 50% of a maximum scavenging capacity. The results were presented as mean values ± standard deviation (s.d.) of three independent measurements. The stoichiometric factor (SF) was calculated for all compounds using the equation [45,46]:

1 $$\mathrm{SF}=\frac{\left[\mathrm{DPPH}\right]}{\left(2\times {\text{IC}}_{\text{50}}\right)}$$

### 2.3. Cell culturing

In this *in vitro* study, we investigated the influence of **Phe*N*AH**s on two model systems: human colorectal carcinoma HCT-116 and human healthy fibroblast cell line MRC-5. Both cell lines of low passages were purchased from the European collection of authenticated cell cultures (ECACC) and were cultivated in Dulbecco's Modified Eagle Medium (DMEM) (Sigma, D5796) cell culture medium supplemented with 10% foetal bovine serum (Sigma, F4135-500 ML) and 1% penicillin/streptomycin (Sigma, P4333-100 ML) in 75 cm^2^ culture flasks. The cells were maintained according to the standardized procedure in the incubator with humidified atmosphere supplemented with 5% CO_2_ at a physiological temperature of 37°C, and after a few passages and a confluence of about 80%, the cells were used in all *in vitro* experiments (*Laboratory for Bioengineering protocol CB-003*).

### 2.4. Cytotoxicity assay

The ability of synthesized compounds to inhibit the growth of two different cell lines was estimated by a standardized 3-(4,5-dimethylthiazol-2-yl)-2,5-diphenyltetrazolium bromide (MTT) assay (*Laboratory for Bioengineering protocol CB-005*). The approximative number of 10 000 cells per well (in 96-well microplates) were seeded and kept in the incubator for 24 h to enable cell adhesion. After the incubation period, the cells were treated with investigated compounds in the concentration range from 0,1 to 500 µM dissolved in DMEM. Cytotoxic effect was evaluated 24 and 72 h from treatment by following the number of survived cells, thus the cell viability. MTT assay is based on spectrophotometric measurement of reduction rate of 3-(4,5-dimethylthiazol-2-yl)-2,5-diphenyltetrazolium bromide (MTT, Acros Organics, 158990010) to purple formazan crystals subsequently dissolved in DMSO (Fisher Chemical, D/4121/PB15) on a microplate reader (Rayto 2100C) at 550 nm. The percentage of the survived (viable) cells was estimated by dividing the absorbance of the treatment with the absorbance of the control (non-treated) cells and multiplied by 100 [47]. As positive controls, Leucovorin and Irinotecan were selected as compounds used in colon cancer treatment.

### 2.5. Statistical analyses

Biological activity was the result of two individual experiments, performed in triplicate for each dose. Statistical analyses were determined using the one-way analysis of variance (ANOVA) test for multiple comparisons, SPSS (Chicago, IL) statistical software package (SPSS for Windows, v. 17, 2008). The IC_50_ values were calculated from the dose curves by a computer program (CalcuSyn). IC_50_ values represent the half-maximal inhibitory concentration of tested compounds to measure the influence in inhibition of cell growth for 50%. An anti-cancer drug candidate must possess a certain selectivity towards cancer in relation to healthy cells. At the beginning of the process of selecting the anti-cancer drug candidate, it is necessary to make a rough preselection between the tested substances. *In vitro* analysis on cancer and healthy cell lines is quantified in different ways, selectivity also. The selectivity index (SI) of tested drugs usually refers to the simple ratio of IC_50_ values determined for cancer and healthy cells [48,49]. Also, the SI can be used for the expression of many other examples, such as SI between antibacterial and antiviral effect, discrimination of the drugs effect between many cancer cell lines, etc. In our experimentation, the values greater than ‘1’ indicate selectivity of the tested substance toward cancer cell line.

### 2.6. Density functional theory (DFT) calculations

All calculations were performed using Gaussian 09 program package [50]. The equilibrium geometries of all **Phe*N*AH**s, as well as all radical species that participate in the reaction mechanism, were calculated using B3LYP functional in conjunction with the 6–311 + g(d,p) basis set [51–53]. Vibrational analysis was performed to confirm the local minima of all compounds (no imaginary frequencies were found). The optimized geometries in the gas phase were used for the simulation of IR spectra (electronic supplementary material, figures S41–S45). IR bands were scaled using the scaling factor obtained using the least-squares method and amount 0.98. IR spectra were prepared using half-width at half-height 4 cm^−1^. The conductor-like polarizable continuum model (CPCM) implemented in Gaussian 09 was used for calculations in different solvents [54]. Calculations in DMSO as solvent were performed for simulation of NMR shifts of all hydrogen and carbon atoms relative to tetramethylsilane (TMS), using the Gauge-Independent Atomic Orbital (GIAO) method. Methanol was selected for the time-dependent density functional theory (TD-DFT) simulation of UV-Vis spectra (half-width at half-height 8 nm, Lorentzian lineshape), as well as for the prediction of free radical scavenging mechanism since it was used as a solvent in experimental assays. Calculations in water were performed to simulate the polar surroundings of the living cell, whereas benzene was used to mimic the nonpolar environment. Charges/multiplicities of the investigated compounds were assigned as follows: charge = 0/multiplicity = 1 for neutral molecules; charge = −1/multiplicity = 1 for anions; charge = 0/multiplicity = 2 for radicals; and charge = 1/multiplicity = 2 for radical cations. To predict the free radical scavenging activity of the investigated compounds, bond dissociation enthalpy (BDE), ionization potential (IP), proton affinity (PA), proton dissociation enthalpy (PDE), and electron transfer enthalpy (ETE) were calculated following the equations (2–6):

2 $$\;\mathrm{BDE}=H(PheNAH\text{–}O^{∙})+H(H^{∙})\text{–}H(PheNAH\text{–}\mathrm{OH})$$

3 $$\;\mathrm{IP}=H(PheNAH\text{–}{\mathrm{OH}}^{∙+})+H(e^{\text{–}})\text{–}H(PheNAH\text{–}\mathrm{OH})$$

4 $$\mathrm{PDE}=H(PheNAH\text{–}O^{∙})+H(H^{+})\text{–}H(PheNAH\text{–}{\mathrm{OH}}^{∙+})$$

5 $$\;\mathrm{PA}=H(PheNAH\text{–}O^{\text{–}})+H(H^{+})\text{–}H(PheNAH\text{–}\mathrm{OH})$$

6 $$\;\;\;\;\;\mathrm{ETE}=H(PheNAH\text{–}O^{∙})+H(e^{\text{–}})\text{–}H(PheNAH\text{–}O^{\text{–}})$$

Reaction enthalpies defined with equations 7–12 were calculated at 298 K [55]. The solvation enthalpy of proton and electron were taken from literature data [56]. The radical stability was evaluated using stabilization energy calculations (Δ*E*_iso_) following the equation 13 [55]:

7 $$\Delta_{r}H_{BDE}=[H(PheNAH\text{–}O^{∙})+H(\mathrm{ROH})]\text{–}[H(PheNAH\text{–}\mathrm{OH})+H({\mathrm{RO}}^{∙})]$$

8 $$\Delta_{r}H_{IP}=[H(PheNAH\text{–}{\mathrm{OH}}^{∙+})+H({\mathrm{RO}}^{\text{–}})]\text{–}[H(PheNAH\text{–}\mathrm{OH})+H({\mathrm{RO}}^{∙})]$$

9 $$\Delta_{r}H_{PDE}=[H(PheNAH\text{–}O^{∙})+H(\mathrm{ROH})]\text{–}[H(PheNAH\text{–}{\mathrm{OH}}^{∙+})+H({\mathrm{RO}}^{\text{–}})]$$

10 $$\Delta_{r}H_{PA}=[H(PheNAH\text{–}O^{-})+H(\mathrm{ROH})]\text{–}[H(PheNAH\text{–}\mathrm{OH})+H({\mathrm{RO}}^{\text{–}})]$$

11 $$\Delta_{r}H_{ETE}=[H(PheNAH\text{–}O^{∙})+H({\mathrm{RO}}^{\text{–}})]\text{–}[H(PheNAH\text{–}O^{-})+H({\mathrm{RO}}^{∙})]$$

12 $$\Delta_{r}H_{BDE}=\Delta_{r}H_{IP}+\Delta_{r}H_{PDE}=\Delta_{r}H_{PA}+\Delta_{r}H_{ETE}$$

13 $$\;\;\Delta E_{\mathrm{iso}}=(H(PheNAH\text{–}O^{∙})+H(\mathrm{Ph}\text{–}\mathrm{OH}))\text{–}(H(PheNAH\text{–}\mathrm{OH})+H(Ph\text{–}O^{∙}))$$

## 3. Results and discussion

### 3.1. Synthesis of phenolic *N*-acylhydrazones (Phe*N*AHs)

**Scheme 1.**
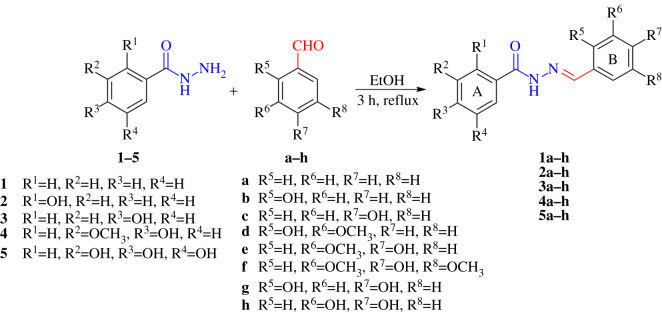
Synthesis of the five series of **Phe*N*AH**s.

**Phe*N*AH**s were synthesized according to the procedure outlined in scheme 1. The benzohydrazides **1**–**5** in the reaction with different aromatic aldehydes **a–h** produced five series of **Phe*N*AH** derivatives. Series **1** of **Phe*N*AH**s was obtained from benzohydrazide, while **Phe*N*AH** series **2**–**5** were obtained from different hydroxybenzohydrazides (2-hydroxybenzohydrazide, 4-hydroxybenzohydrazide, 4-hydroxy-3-methoxybenzohydrazide and 3,4,5-trihydroxybenzohydrazide, respectively). These reactions were performed in ethanol by heating for 3 h and without any catalyst. All **Phe*N*AH** products were isolated by precipitation and filtration, in moderate to excellent yield (49–98%), scheme 1. Moderate yield (49–68%) was achieved for most derivatives of series **1** (**1a**–**c**, **1g** and **1h**) and compounds **3b**, **3h**, and **5a**. All other **Phe*N*AH** derivatives were obtained in good to excellent yield. It is important to emphasize that within these five series of **Phe*N*AH** derivatives, a total of 40 compounds were synthesized. The **Phe*N*AH**s were characterized experimentally and theoretically by ^1^H NMR, ^13^C NMR, UV-Vis and FT-IR spectra. The purity of all obtained compounds was determined by HPLC analysis (greater than 95%, electronic supplementary material, tables S91–S130 and figures S121–S160).

### 3.2. IR spectral characterization

All **Phe*N*AH**s were characterized using IR spectroscopy. To confirm the assignation of experimental bands, IR spectra were simulated using Density Functional Theory (DFT). The IR spectra of all compounds are provided in the electronic supplementary material, figures S46–S55. Excellent agreement between experimental and modelled spectra was achieved. The bands assigned to O–H (except for **1a**) and N–H stretching vibrations are present in all experimental spectra from 3600–3200 cm^−1^. The bands in the 3100–3000 cm^−1^ region originate from aromatic C–H stretching vibrations, while those in the region 3000–2800 cm^−1^ correspond to aliphatic C–H vibrations (**d**, **e**, and **f** derivatives). The bands assigned to C=O and C=N stretching vibrations were identified from 1650–1600 cm^−1^. The bands close to 1600 cm^−1^ and 1450 cm^−1^ were assigned to aromatic C–C vibrations, while those around 1500 cm^−1^ to bending H–C=C. On the other hand, the bands located around 1550 cm^−1^ were assigned to bending H–N–N deformational vibration, while those near 1360 cm^−1^ to H–C=N bending. In the 1330–1100 cm^−1^ region, bands that correspond to the C–O, Ar–C–C=O and N–N stretching vibrations were identified. It is important to emphasize that in several cases, the absence of some bands (H–C=C, H–N–N and H–C=N) or overlapping of the bands (such as C=O and C=N or O–H and N–H) was observed (spectral data and electronic supplementary material, figures S46–S55),

### 3.3. UV-Vis spectral characterization

**Phe*N*AH**s were characterized by UV-Vis experimentally and theoretically. Modelled spectra were acquired using TD-DFT, and excellent agreement with experimental spectra was achieved. Kohn-Sham orbitals were constructed to identify the segments of the molecule responsible for electronic transitions (electronic supplementary material, figures S66–S108; isovalues = 0.02 e au^−3^). UV-Vis spectra of all derivatives are given in the supplementary material (electronic supplementary material, figures S56–S65;). For all compounds, one major absorption band appeared in the 300–350 nm region. This experimental band is identified as a result of HOMO (highest occupied molecular orbital) to LUMO (lowest unoccupied molecular orbital) electronic transition. On the other hand, another major band appeared around 210 nm. Electronic transitions responsible for this band differ from compound to compound, depending on aromatic rings substitutions. Hence, these transitions responsible for the appearance of the particular bands are presented in table 1 and electronic supplementary material, tables S1–S4.

**Table 1.** Electronic transitions responsible for experimental bands for compounds **5a–h.**

**5a**			**5b**			**5c**			**5d**		
transition		*λ*(nm)	transition		*λ*(nm)	transition		*λ*(nm)	transition		*λ*(nm)
HOMO	LUMO	308.5	HOMO	LUMO	327.5	HOMO	LUMO	320	HOMO	LUMO	341
HOMO-1	LUMO		HOMO-3	LUMO	299.5	HOMO-1	LUMO+1	213	HOMO-1	LUMO	310.5
HOMO-3	LUMO	213.5	HOMO-1	LUMO		HOMO-3	LUMO		HOMO-2	LUMO	297.5
HOMO	LUMO+2		HOMO-3	LUMO	290	HOMO	LUMO+2		HOMO-6	LUMO	213
HOMO-1	LUMO+2		HOMO	LUMO+1		HOMO-2	LUMO+2		HOMO-5	LUMO	
HOMO	LUMO+3		HOMO-3	LUMO+1	213.5	HOMO-1	LUMO+3		HOMO-1	LUMO+2	
HOMO-2	LUMO+1		HOMO	LUMO+2		HOMO-1	LUMO+6		HOMO	LUMO+9	
HOMO-1	LUMO+3		HOMO-1	LUMO+2		HOMO	LUMO+9		HOMO-1	LUMO+2	
HOMO-3	LUMO+1		HOMO-1	LUMO+3		HOMO-2	LUMO+3		HOMO	LUMO+4	
HOMO-2	LUMO+2		HOMO-3	LUMO+2		HOMO	LUMO+8		HOMO-4	LUMO+1	
HOMO-2	LUMO+3		HOMO	LUMO+8					HOMO-2	LUMO+2	
HOMO	LUMO+6		HOMO-2	LUMO+3					HOMO-3	LUMO+2	
HOMO-1	LUMO+5		HOMO	LUMO+7					HOMO-2	LUMO+4	

**5e**			**5f**			**5g**			**5h**		
transition		*λ*(nm)	transition		*λ*(nm)	transition		*λ*(nm)	transition		*λ*(nm)
HOMO	LUMO	327.5	HOMO	LUMO	328.5	HOMO	LUMO	332	HOMO	LUMO	328.5
HOMO-1	LUMO	301	HOMO-4	LUMO	242	HOMO-3	LUMO	302.5	HOMO-3	LUMO	303.5
HOMO-3	LUMO	288	HOMO-3	LUMO+1		HOMO-1	LUMO		HOMO-1	LUMO	
HOMO	LUMO+1		HOMO-2	LUMO+1	212	HOMO-3	LUMO	289.5	HOMO-3	LUMO	292
HOMO-5	LUMO	212.5	HOMO	LUMO+4		HOMO	LUMO+1		HOMO-3	LUMO	236
HOMO-4	LUMO		HOMO-5	LUMO+1		HOMO-3	LUMO	242.5	HOMO	LUMO+2	
HOMO-3	LUMO+1		HOMO-1	LUMO+2		HOMO	LUMO+2		HOMO-5	LUMO+1	213
HOMO-1	LUMO+2		HOMO-3	LUMO+2		HOMO-2	LUMO+1	212	HOMO-4	LUMO+1	
HOMO-1	LUMO+3					HOMO-1	LUMO+3		HOMO-3	LUMO+2	
HOMO-5	LUMO+1					HOMO-3	LUMO+2		HOMO	LUMO+8	
HOMO	LUMO+9					HOMO	LUMO+7		HOMO-2	LUMO+3	
HOMO	LUMO+10					HOMO-2	LUMO+3		HOMO-1	LUMO+1	
HOMO-2	LUMO+3					HOMO-2	LUMO+4				
HOMO	LUMO+7										

Bands that correspond to HOMO–LUMO transitions for compounds **5a–h** were in the range of 308–341 nm, electronic supplementary material, figures S56–S65. In simulated spectra, these bands are redshifted for 10–20 nm. In the case of **5a**, HOMO-1 to LUMO transition also contributes to this absorption band. Moreover, in the spectra of other analogues of series **5**, additional bands were noted. In the case of **5b**, HOMO-3 to LUMO is mainly responsible for the absorption band at 290 nm, while the band at 299.5 nm originates primarily from HOMO-1 to LUMO electronic transition. For compound **5d**, the band at 297.5 nm is assigned to HOMO-2 to LUMO, while the band at 310.5 nm corresponds to HOMO-1 to LUMO transition. On the other hand, for compound **5e,** the band at 288 nm originates from HOMO to LUMO+1 and HOMO-3 to LUMO transitions, while the band at 301 nm corresponds to HOMO-1 to LUMO transition. In the case of **5f**, the band at 242 nm is a consequence of HOMO-4 to LUMO. Furthermore, three additional bands were observed in the spectra of **5g** and **5h** at similar positions. For analogue **5g**, absorption at 242.5 nm is a consequence of the HOMO to LUMO+2, the band at 289.5 nm originates mainly from HOMO-3 to LUMO, whereas the band at 302.5 is assigned to HOMO-1 to LUMO transition. Similarly, for compound **5h**, the band at 236 nm corresponds to HOMO to LUMO+2 and HOMO-3 to LUMO, the absorption at 292 nm originates from HOMO-3 to LUMO, while the band at 303.5 nm is assigned to HOMO-1 to LUMO with a minor contribution of HOMO-3 to LUMO transition. Electronic transitions responsible for experimental bands for all other compounds are provided in electronic supplementary material, tables S1–S4.

### 3.4. NMR spectral characterization of PheNAHs

All compounds were characterized using experimental and theoretical ^1^H NMR and ^13^C NMR spectra. NMR spectra of all **Phe*N*AHs** are presented in the electronic supplementary material, figures S1–S40. Simulated spectra showed good agreement with experimentally obtained data. Generally, ^1^H NMR spectra of **Phe*N*AH**s consist of peaks originating from aromatic protons, imine proton (H–C=N), hydroxy proton(s), and proton from the –NH group. Additionally, spectra of derivatives **d**, **e**, and **f** displayed signals which correspond to the protons from methoxy groups. Signals which correspond to aromatic protons were mainly observed in the 8.5–6.3 ppm area, resonating mostly as doublets, multiplets or doublet of doublets. Moreover, imine proton, as well as proton attached to *N*-atom, resonated as sharp singlets close to 8.5 ppm (H–C = N) and in the 12.0–11.0 ppm region (N–H). On the other hand, signals related to hydroxy protons vary from compound to compound. These peaks were observed in the 10.0–9.0 ppm and/or 12.0–11.0 ppm region as sharp or broad singlets. Finally, ^1^H NMR peaks that correspond to methoxy groups appeared as sharp singlets around 3.80 ppm. NMR spectral data for all **Phe*N*AH**s is provided in the electronic supplementary material.

Similarly, ^13^C NMR spectra displayed peaks that correspond to aromatic carbons, iminic carbon and carbons from carbonyl and methoxy groups. Generally, carbonyl and iminic signals were observed at the highest chemical shifts, both in experimental and simulated spectra. On the other hand, peaks originating from methoxy carbons were located close to 60 ppm. Modelled spectra distinguished substituted aromatic carbons from non-substituted, indicating the higher chemical shifts of substituted ones. NMR spectral data for all **Phe*N*AH**s is provided in the electronic supplementary material.

### 3.5. DPPH radical scavenging activity

The assessment of the radical scavenging activity of **Phe*N*AH**s was performed *in vitro* using the 2,2-diphenyl-1-picrylhydrazyl (DPPH) assay. This method was chosen since it is considered credible for the prediction of the antioxidant capacity against reactive oxygen species present in the living cell [57,58]. Nordihydroguaiaretic acid (NDGA) and quercetin were used as reference compounds due to their well-known radical scavenging potency. Among 40 tested compounds, 20 derivatives expressed antioxidant activity against DPPH radical, table 2. The antioxidant screening revealed the best interaction of the compounds **5a–h** with DPPH radical.

**Table 2.** Experimental IC_50_ (μM) values for inactivation of DPPH free radical for compounds **1**–**5**.

**compound**	**1a**	**1b**	**1c**	**1d**	**1e**	**1f**	**1g**	**1h**
IC_50_	>100	>100	>100	>100	31.4 ± 0.9	17.2 ± 1.1	>100	2.93 ± 0.1
SF	/	/	/	/	0.4	0.7	/	4.3
**compound**	**2a**	**2b**	**2c**	**2d**	**2e**	**2f**	**2g**	**2h**
IC_50_	>100	>100	>100	>100	20.1 ± 0.9	6.5 ± 0.4	>100	1.6 ± 0.0
SF	/	/	/	/	0.6	1.9	/	7.8
**compound**	**3a**	**3b**	**3c**	**3d**	**3e**	**3f**	**3g**	**3h**
IC_50_	>100	>100	>100	>100	28.4 ± 0.2	16.6 ± 0.2	>100	2.5 ± 0.1
SF	/	/	/	/	0.4	0.8	/	5
**compound**	**4a**	**4b**	**4c**	**4d**	**4e**	**4f**	**4g**	**4h**
IC_50_	>100	>100	>100	>100	22.1 ± 1.1	6.3 ± 0.3	>100	3.4 ± 0.1
SF	/	/	/	/	0.6	2	/	3.7
**compound**	**5a**	**5b**	**5c**	**5d**	**5e**	**5f**	**5g**	**5h**
IC_50_	1.3 ± 0.1	1.2 ± 0.1	2.9 ± 0.1	1.0 ± 0.1	1.9 ± 0.1	1.1 ± 0.1	0.9 ± 0.1	0.7 ± 0.1
SF	9.6	10.4	4.3	12.5	6.6	11.4	13.9	17.9
**compound**	**NDGA**	**Quercetin**						
IC_50_	1.7 ± 0.1	1.9 ± 0.1						
SF	7.4	6.6						

The IC_50_ values for derivatives **5a–h** were in the 0.7–2.9 µM range, where derivative **5h** exhibited the best radical scavenging ability (IC_50_ = 0.7 ± 0.1 µM). On the other hand, **h** analogues of the series **1**–**4** exposed excellent antiradical potency (1.6–3.4 µM), while **e** and **f** derivatives of these series expressed noteworthy scavenging activity (table 2 and electronic supplementary material, tables S5–S9). In addition, stoichiometric factor (SF) was calculated for all compounds (table 2). Namely, SF presents one of the ways to express the antioxidant capability of compounds. The compound is considered as a good antioxidant if SF ≥ 2. Therefore, the higher value of SF implies better scavenging ability. Based on the obtained values, all **Phe*N*AH**s of series **5** can be considered as excellent antioxidants with SF values ranging from 4.3 to 17.9. Also, the SF of all **h** analogues of series **1–4** is greater than or equal to 2, implying their good antioxidant capacity.

Excellent scavenging activity of compounds **5** and of **h** analogues of **1**–**4** can be attributed to the favourable substitution of rings A and B. It is well-known that the antioxidant capacity of phenolic compounds depends on the type, number, and position of neighbouring groups such as –OH, –OR, –NH_2_ to the phenolic hydroxy group [59]. Their presence increases the stability of the formed phenoxy radical through resonance and electron-donating effects. Moreover, the hydrogen bond occurs between phenoxy radical and –OH group in catechol- and pyrogallol-like compounds, resulting in even better stabilization of produced phenoxy radical [60–62]. These statements are in excellent agreement with obtained results since all compounds containing pyrogallol and/or catechol units (**5a**–**h**, and all **h** analogues) expressed exceptional scavenging activity. Compounds obtained from vanillin and syringaldehyde (analogues **e** and **f**) also exhibited noticeable scavenging activity toward DPPH. These compounds bear methoxy group(s) next to the phenolic hydroxy group, and their influence on the scavenging activity was especially observed in series **1–4**. On the other hand, low activity of other derivatives (**a–d** and **g**) of the **1–4** series is a consequence of insufficient stabilization of phenoxy radical.

### 3.6. Electronic properties of Phe*N*AHs

The chemical reactivity of **Phe*N*AH**s toward free radical species was estimated by mutual comparison of E_HOMO_ and E_LUMO_ for each analogue. Graphical interpretations of HOMOs and LUMOs are provided in supplementary material, figures S66–S108. The high values of E_HOMO_ indicate good electron-donating ability which is considered an important factor for radical scavenging [63]. Furthermore, the HOMO–LUMO gap describes the chemical reactivity of the molecule, where a lower energy barrier indicates the higher possibility of a reaction with free radicals. Reactivity toward free radicals is also influenced by the stability of generated radicals from the compound after radical scavenging. Therefore, stabilization energies (Δ*E*_iso_) were calculated for radicals at specified positions to estimate the involvement of groups in antioxidant activity. It is important to point out that these electronic parameters are fully comparable between active and non-active compounds within the same series.

The electronic properties of compounds **1a–h** calculated in methanol are presented in table 3, while the results obtained in water and benzene are given in electronic supplementary material, table S10. Excellent agreement between experimental IC_50_ and theoretical data was achieved. Namely, the highest E_HOMO_ values were obtained for derivatives **1e**, **1f** and **1h** (−0.221, −0.216, and −0.223 eV, respectively) which correspond to results obtained in the DPPH assay. The highest E_HOMO_ was observed for compound **1f**, which is explained by the presence of two -OCH_3_ groups on the B-ring with electron-donating effects. Furthermore, low energy values of the HOMO–LUMO gap were obtained for these analogues (table 3), indicating their increased reactivity toward DPPH radical. The lowest Δ*E*_iso_ values were also noted for compounds **1e**, **1f** and **1h**, implying the best radical stabilization after radical scavenging. It is important to emphasize that stabilization energies were separately calculated for the formation of N**^•^** and O**^•^** radicals. The highest involvement of the B-ring R^7^-OH groups was observed since the lowest Δ*E*_iso_ values were calculated for R^7^-O**^•^** radical formation (table 3). These results revealed enhanced reactivity of **1e**, **1f** and **1h** analogues toward DPPH radical in comparison to derivatives **1a–d** and **1g**. Electronic properties of derivatives **1a–d** and **1g** correspond to their poor radical scavenging activity in the DPPH assay.

**Table 3.** Calculated electronic properties of compounds **1a–h** in methanol.

compound		HOMO (eV)	LUMO (eV)	HOMO-LUMO gap (eV)	Δ*E*_iso_ (kJ mol^−1^)
methanol					
**1a**	NH	−0.235	−0.074	0.161	60.961
**1b**	R^5^-OH (B)	−0.233	−0.078	0.155	22.653
	NH				18.258
**1c**	R^7^-OH (B)	−0.226	−0.073	0.153	−18.392
	NH				15.787
**1d**	R^5^-OH (B)	−0.224	−0.077	0.147	−8.588
	NH				15.588
**1e**	R^7^-OH (B)	−0.221	−0.073	0.148	−25.932
	NH				13.818
**1f**	R^7^-OH (B)	−0.216	−0.073	0.143	−43.310
	NH				11.011
**1g**	R^5^-OH (B)	−0.225	−0.074	0.151	21.038
	R^7^-OH (B)				−11.707
	NH				8.357
**1h**	R^6^-OH (B)	−0.223	−0.073	0.150	−35.221
	R^7^-OH (B)				−46.072
	NH				15.884

Generally, for all other compounds, similar results were obtained. Electronic properties of all other derivatives are provided in the electronic supplementary material, tables S11–S18. Compounds that expressed antioxidant activity from series **2–4** were also **e**, **f** and **h Phe*N*AH** derivatives. As in the cases of compounds **1e**, **1f** and **1h**, the highest E_HOMO_, low HOMO-LUMO gap, and the lowest Δ*E*_iso_ values of the B-ring R^7^-OH group were observed for **e**, **f** and **h** analogues from series **2–4**. Furthermore, all compounds from series **5** displayed exceptional activity toward DDPH radical. The electronic properties calculated for derivatives **5a–h** are presented in table 4. Since all derivatives **5** were active toward DPPH, smaller but noticeable differences were observed by comparison of their electronic properties. The most favourable electronic parameters for radical scavenging were also noted for derivatives **5e**, **5f** and **5h** (table 4), which agrees with their experimental IC_50_ values. Moreover, for compounds **5a–h**, the obtained results suggested the involvement of all ring A –OH groups, especially the R^3^-OH group. In the case of **5h**, the influence of the B-ring –OH groups was also noted according to their calculated Δ*E*_iso_ values (table 4).

**Table 4.** Calculated electronic properties of compounds **5a–h** in methanol.

compound		HOMO (eV)	LUMO (eV)	HOMO-LUMO gap (eV)	Δ*E*_iso_ (kJ mol ^−1^)
methanol					
**5a**	R^2^-OH (A)	−0.233	−0.076	0.157	−21.600
	R^3^-OH (A)				−47.419
	R^4^-OH (A)				−24.7532
	NH				20.613
**5b**	R^2^-OH (A)	−0.230	−0.076	0.154	−20.891
	R^3^-OH (A)				−46.561
	R^4^-OH (A)				−23.871
	R^5^-OH (B)				24.866
	NH				13.831
**5c**	R^2^-OH (A)	−0.224	−0.071	0.153	−22.141
	R^3^-OH (A)				−48.249
	R^4^-OH (A)				−25.352
	R^7^-OH (B)				−19.350
	NH				12.311
**5d**	R^2^-OH (A)	−0.224	−0.076	0.148	−20.896
	R^3^-OH (A)				−46.624
	R^4^-OH (A)				−21.400
	R^5^-OH (B)				−5.991
	NH				14.159
**5e**	R^2^-OH (A)	−0.220	−0.071	0.149	−21.915
	R^3^-OH (A)				−48.133
	R^4^-OH (A)				−25.102
	R^7^-OH (B)				−26.560
	NH				10.124
**5f**	R^2^-OH (A)	−0.215	−0.075	0.140	−24.593
	R^3^-OH (A)				-48.199
	R^4^-OH (A)				−24.9081
	R^7^-OH (B)				−43.8669
	NH				7.690
**5g**	R^2^-OH (A)	−0.223	−0.071	0.152	−21.211
	R^3^-OH (A)				−47.327
	R^4^-OH (A)				−24.270
	R^5^-OH (B)				23.307
	R^7^-OH (B)				−12.592
	NH				4.750
**5h**	R^2^-OH (A)	−0.221	−0.071	0.150	−21.923
	R^3^-OH (A)				−47.973
	R^4^-OH (A)				−25.344
	R^6^-OH (B)				−35.557
	R^7^-OH (B)				−46.700
	NH				12.503

### 3.7. Antioxidant mechanism investigations

The thermodynamical approach was used to estimate the most possible radical scavenging mechanism of **Phe*N*AH** derivatives. Phenolic antioxidants entrap radical species by several plausible mechanisms: hydrogen atom transfer (HAT), single-electron transfer-proton transfer (SET-PT), and sequential proton loss electron transfer (SPLET) [64]. All these pathways have the same outcome, i.e. the inactivation of free radical species and the formation of the corresponding phenoxy radical from the antioxidant. In the HAT pathway, the hydrogen atom is directly transferred to the radical [65]. Herein, the bond dissociation enthalpy (BDE) is the parameter that illustrates the probability of the HAT mechanism, since the homolytic cleavage of the phenolic O–H bond is required. The SET-PT is a two-step route that is initiated with electron transfer to the radical species, resulting in the formation of the radical-cationic antioxidant [66]. In the second step, radical cation deprotonation occurs, after which the phenoxy radical is formed [66]. For the SET-PT, ionization potential (IP) and proton dissociation enthalpy (PDE) values describe the preferability of this radical scavenging pathway. On the other hand, the SPLET pathway starts with the deprotonation of antioxidant, whereas in the second step an electron is moved to the radical from anion [67]. Therefore, proton affinity (PA) and electron transfer enthalpy (ETE) values designate the possibility of the SPLET route. Bearing this in mind, the general evaluation of the antioxidant pathway in the absence of free radicals was achieved by mutual comparison of BDE, IP, PDE, PA and ETE values [66]. To investigate the impact of solvents' polarity, all calculations were performed in methanol, water and benzene. Calculated thermodynamic parameters for compounds **5a–h** in methanol are presented in table 5, while parameters obtained in water and benzene, as well as the results for **Phe*N*AH**s **1–4,** are provided in the electronic supplementary material, tables S19–S31.

**Table 5.** Calculated thermodynamic parameters (kJ mol^−1^) of compounds **5a–h** in methanol.

		HAT	SET-PT		SPLET	
		BDE	IP	PDE	PA	ETE
methanol						
**5a**	R^2^-OH (A)	331	506	−14	123	370
	R^3^-OH (A)	305		−40	101	366
	R^4^-OH (A)	328		−17	122	367
	NH	373		28	162	373
**5b**	R^2^-OH (A)	332	498	−5	122	372
	R^3^-OH (A)	306		−31	99	369
	R^4^-OH (A)	329		−8	121	370
	R^5^-OH (B)	377		40	176	363
	NH	366		29	145	383
**5c**	R^2^-OH (A)	330	478	14	123	368
	R^3^-OH (A)	304		−12	102	364
	R^4^-OH (A)	327		11	123	366
	R^7^-OH (B)	333		17	137	358
	NH	365		49	166	361
**5d**	R^2^-OH (A)	332	480	13	124	369
	R^3^-OH (A)	306		−12	102	366
	R^4^-OH (A)	331		13	123	369
	R^5^-OH (B)	346		28	161	347
	NH	367		48	145	383
**5e**	R^2^-OH (A)	331	468	24	124	368
	R^3^-OH (A)	304		−3	102	364
	R^4^-OH (A)	327		21	123	366
	R^7^-OH (B)	326		19	146	342
	NH	363		56	163	361
**5f**	R^2^-OH (A)	328	456	33	123	366
	R^3^-OH (A)	304		10	102	364
	R^4^-OH (A)	328		33	122	367
	R^7^-OH (B)	309		14	144	326
	NH	360		65	165	357
**5g**	R^2^-OH (A)	331	478	15	122	370
	R^3^-OH (A)	305		−11	100	367
	R^4^-OH (A)	328		12	121	369
	R^5^-OH (B)	376		59	173	365
	R^7^-OH (B)	340		23	135	366
	NH	357		41	148	371
**5h**	R^2^-OH (A)	331	474	18	123	369
	R^3^-OH (A)	304		−8	102	364
	R^4^-OH (A)	327		15	123	366
	R^6^-OH (B)	317		5	128	351
	R^7^-OH (B)	306		−6	118	350
	NH	365		53	165	362

The obtained results revealed that the SET-PT mechanism can be excluded in all cases and all solvents due to significantly higher values of IP in comparison to BDE and PA (table 5, electronic supplementary material, tables S19–S31). On the other hand, in methanol and water, the PA values are noticeably lower than BDE, indicating the SPLET mechanism is thermodynamically favourable in polar solvents. The results obtained in benzene indicate that HAT-SPLET competition should be considered. Here, small differences between BDE and PA values were observed (table 5, electronic supplementary material, tables S19–S31). It is important to emphasize that these parameters were also calculated separately for −NH and each of the -OH groups in the molecules. Here, for compounds **5a–h**, the lowest values of BDE and PA were found for the R^3^-OH (ring A), which is an additional confirmation of its high involvement in the antioxidant activity (table 5). A similar trend was observed for compounds **1–4** (electronic supplementary material, tables S19–S30).

The estimation of the most preferable radical scavenging pathway was performed in the presence of radical species (table 6 and electronic supplementary material, tables S32–S90). Namely, seven medically relevant radicals and DPPH were selected for the prediction of the reaction course with **Phe*N*AH**s (table 6). The selection was made based on their appearance and behaviour in the living cell [68]. The influence of different solvents, as well as the electronic properties of selected radicals, were taken into account [69]. These calculations were performed with all compounds that exhibited antioxidant activity. The results obtained in methanol for the most active compound **5h** are presented in table 6, while the results obtained in water and benzene as solvents are provided in the electronic supplementary material, tables S89 and S90. Here, the calculated values of Δ*H*_BDE_, Δ*H*_IP_ and Δ*H*_PDE_, Δ*H*_PA_, and Δ*H*_ETE_ were mutually compared for the reaction with each radical. The SET-PT pathway can be eliminated in all solvents due to the high values of Δ*H*_IP_ (table 6, electronic supplementary material, tables S89 and S90). In the polar solvents similar values for Δ*H*_BDE_ and Δ*H*_PA_ were observed, indicating the competition between the HAT and SPLET mechanisms (table 6 and electronic supplementary material, table S89). The HAT mechanism prevails slightly in the reaction with HO**^•^** radical, whereas the SPLET pathway prevails in the cases with HOO**^•^** and CH_3_OO**^•^** radicals. On the other hand, the results obtained in benzene showed that the SPLET mechanism is mainly dominant, except in the cases of HO**^•^** and DPPH radicals where HAT-SPLET competition was observed (electronic supplementary material, table S90). It is important to emphasize that compound **5h** possesses multiple groups for engaging radical species, therefore, all possibilities were considered. Calculated data showed the most favourable involvement of the R^3^-OH and R^7^-OH groups, with a significant contribution of R^2^-OH and R^4^-OH groups. Similar results were obtained for other derivatives of series **5** (electronic supplementary material, tables S68–S88). The lowest energy values for analogues **5a–g** were observed for the R^3^-OH group. It is worth pointing out that in the case of **5f** the contribution of the R^7^-OH to radical scavenging activity can't be neglected (electronic supplementary material, tables S83–S85).

**Table 6.** Calculated reaction enthalpies (kJ mol^−1^) for the reactions of compound **5h** with selected radials in methanol.

5 h	IC_50_ (µM) = 0.7 ± 0.1					
		HAT	SET-PT		SPLET	
radical	position	Δ*H*_BDE_	Δ*H*_IP_	Δ*H*_PDE_	Δ*H*_PA_	Δ*H*_ETE_
^.^OCH_3_	R^2^-OH (A)	−89	130	−219	−114	25
	R^3^-OH (A)	−115		−245	−136	21
	R^4^-OH (A)	−92		−222	−115	23
	R^6^-OH (B)	−102		−233	−110	7
	R^7^-OH (B)	−113		−244	−120	6
	NH	−54		−185	−73	19
^.^OC(CH_3_)_3_	R^2^-OH (A)	−97	130	−227	−122	25
	R^3^-OH (A)	−123		−253	−144	21
	R^4^-OH (A)	−100		−230	−123	23
	R^6^-OH (B)	−110		−241	−117	7
	R^7^-OH (B)	−121		−252	−128	6
	NH	−62		−192	−81	18
^.^OH	R^2^-OH (A)	−158	57	−216	−110	−48
	R^3^-OH (A)	−185		−242	−132	−52
	R^4^-OH (A)	−162		−219	−111	−51
	R^6^-OH (B)	−172		−229	−106	−66
	R^7^-OH (B)	−183		−240	−116	−67
	NH	−124		−181	−69	−55
^.^OOH	R^2^-OH (A)	−21	154	−175	−70	49
	R^3^-OH (A)	−47		−201	−91	45
	R^4^-OH (A)	−24		−178	−71	47
	R^6^-OH (B)	−34		−188	−65	31
	R^7^-OH (B)	−45		−200	−75	30
	NH	14		−140	−28	42
^.^OOCH_3_	R^2^-OH (A)	−12	164	−176	−71	59
	R^3^-OH (A)	−38		−202	−93	55
	R^4^-OH (A)	−15		−179	−72	56
	R^6^-OH (B)	−26		−190	−66	41
	R^7^-OH (B)	−37		−201	−77	40
	NH	23		−141	−30	52
^.^OO–CH = CH_2_	R^2^-OH (A)	−13	135	−147	−42	30
	R^3^-OH (A)	−39		−174	−64	25
	R^4^-OH (A)	−16		−151	−43	27
	R^6^-OH (B)	−26		−161	−38	12
	R^7^-OH (B)	−38		−172	−48	11
	NH	22		−113	−1	23
DPPH	R^2^-OH (A)	10	89	−79	26	−16
	R^3^-OH (A)	−16		−105	4	−20
	R^4^-OH (A)	7		−82	25	−18
	R^6^-OH (B)	−3		−93	30	−34
	R^7^-OH (B)	−15		−104	20	−35
	NH	45		−45	67	−23
O_2_^.−^	R^2^-OH (A)	63	382	−318	14	49
	R^3^-OH (A)	37		−344	−7	45
	R^4^-OH (A)	60		−322	13	47
	R^6^-OH (B)	50		−332	19	31
	R^7^-OH (B)	39		−343	9	30
	NH	98		−284	55	42

The estimation of the radical scavenging pathways in the presence of harmful radical species was achieved for all other active compounds. In series **1–4** only **e**, **f**, and **h** derivatives exhibited antioxidant activity toward DPPH. For these analogues, HAT-SPLET competition was observed in reactions with all radicals in polar solvents, except for HO**^•^**, where the HAT mechanism is predominant (electronic supplementary material, tables S32–S67). In benzene as a solvent, the SPLET mechanism was mainly observed, whereas HAT-SPLET competition is evident for reactions with HO**^•^** and DPPH radicals (electronic supplementary material, tables S32–S67). Furthermore, the lowest energy values were calculated for the R^7^-OH group for all **e** and **f** derivatives of series **1–4**. It is important to emphasize that in such cases where the SPLET mechanism is considered, the contribution of other groups should not be neglected due to similar values of Δ*H*_PA_. Nevertheless, the Δ*H*_ETE_ values suggested that the second step of the SPLET pathway is much more favourable for the R^7^-OH group. On the other hand, for **h** derivatives, the involvement of R^6^-OH and R^7^-OH was clearly observed.

### 3.8. Cytotoxicity of Phe*N*AHs

The **Phe*N*AH** derivatives were evaluated for their ability to inhibit the growth of HCT-116 and MRC-5 cell lines. The IC_50_ values for all compounds are presented in table 7, while graphical interpretations of the obtained results are provided in electronic supplementary material, figures S109–S118.

**Table 7.** Growth inhibitory effects (IC_50,_ μM) of **Phe*N*AH**s and commercial cytostatic drugs on MRC-5 and HCT-116 cell lines after 24 and 72 h exposure.

**IC_50_**		**1a**	**1b**	**1c**	**1d**	**1e**	**1f**	**1g**	**1h**
**MRC-5**	24 h	>500	>500	>500	>500	>500	>500	>500	>500
	72 h	>500	>500	>500	306.5	>500	>500	>500	>500
**HCT-116**	24 h	>500	249.4	>500	>500	>500	>500	>500	>500
	72 h	>500	139.1	>500	66.6	>500	>500	315.9	>500
		**2a**	**2b**	**2c**	**2d**	**2e**	**2f**	**2g**	**2h**
**MRC-5**	24 h	>500	>500	>500	37.9	>500	>500	>500	>500
	72 h	>500	232.7	>500	87.3	>500	>500	>500	>500
**HCT-116**	24 h	>500	26.9	>500	80.5	>500	>500	>500	>500
	72 h	>500	70.5	>500	26.2	>500	>500	61.2	208
		**3a**	**3b**	**3c**	**3d**	**3e**	**3f**	**3g**	**3h**
**MRC-5**	24 h	>500	>500	>500	>500	>500	>500	>500	>500
	72 h	>500	>500	>500	213.2	>500	>500	439.7	>500
**HCT-116**	24 h	>500	>500	>500	>500	>500	>500	>500	>500
	72 h	>500	101.3	>500	94.1	>500	>500	129.8	106.9
		**4a**	**4b**	**4c**	**4d**	**4e**	**4f**	**4g**	**4h**
**MRC-5**	24 h	>500	>500	>500	>500	>500	>500	>500	>500
	72 h	>500	290	>500	127.6	>500	>500	306.6	>500
**HCT-116**	24 h	>500	249.7	>500	>500	>500	>500	193.5	>500
	72 h	>500	91.4	>500	202.1	>500	>500	>500	>500
		**5a**	**5b**	**5c**	**5d**	**5e**	**5f**	**5g**	**5h**
**MRC-5**	24 h	175.46	104.44	91.99	179.64	>500	84.33	76.35	>500
	72 h	59.24	52.15	31.88	457.52	45.76	40.0	78.6	50.55
**HCT-116**	24 h	247.2	62.6	71.75	89.91	>500	86.92	99.9	>500
	72 h	63.53	32.35	38.45	>500	65.23	43.6	77.34	58.76
		**leucovorin**	**irinotecan**						
**MRC-5**	24 h	>500	>500						
	72 h	179.76	35.19						
**HCT-116**	24 h	>500	>500						
	72 h	>500	100.88						

In almost all groups of compounds, there is a decrease in cell viability, while in some there is no effect, especially on a healthy MRC-5 cell line (with IC_50_ values greater than 500 µM). Available data on different hydrazone derivatives show similar results to ours [70], while some show significantly lower IC_50_ values [71,72]. It is noticeable that the presence of different functional groups, and in different positions, leads to different effects on cancer and healthy cell lines. Here, compounds with specified modifications selectively influence the cancer cell line, showing cytotoxic character. Based on the obtained results, modifications **b**, **d**, **g** and **h** within the tested series stand out (table 7, scheme 1). The modifications **b**, **d** and **g** possess the -OH group in the ortho position (R^5^) of the B ring (scheme 1), indicating its involvement in the enhanced cytotoxic effect in comparison with other B ring modifications. Some authors have also reported a favourable influence of substituents in the ortho position, and explain this with intramolecular H-bond formation between this -OH group and the hydrogen atom of the hydrazone −C=N–NH– moiety [73,74]. On the other hand, the **h** modification comprises two −OH groups in the R^6^ and R^7^ positions, which in series **2, 3** and **5** slightly reduces cell viability. Besides the B ring modification, the substitution of the A ring also influences the cytotoxic effects on both HCT-116 and MRC-5 cell lines. Based on the obtained IC_50_ values, the derivatives **b**, **d** and **g** of series **2** expressed enhanced cytotoxic effect in comparison to those from series **1**, **3** and **4**. Here, the highest activity, i.e. the lowest IC_50_ value, was determined for compound **2b**, which bears the *o*-OH group on both rings. Moreover, analogues **a**, **c**, **e** and **f** of series **1–4** showed no activity, while those of series **5** expressed cytotoxic effect, indicating the influence of A ring substitution on cell lines.

It is important to discuss the selectivity of **Phe*N*AH**s between two model systems, i.e. between healthy and cancer cell lines. Generally, modification **b** stands out, expressing a good cytotoxic effect, as well as noticeable selectivity in almost every series both after 24 and after 72 h (table 8).

**Table 8.** Selectivity index of modification **b** and commercial cytostatic drugs after 24 and 72 h exposure.

SI	**1b**	**2b**	3b	**4b**	**5b**	leucovorin	irinotecan
24 h	>2.01	>18.59	1.00	>2.00	1.67	1.00	1.00
72 h	>3.59	3.30	>4.94	3.17	1.61	<0.36	0.35

In the series **1–3**, analogues **d** possess the strongest cytotoxic effect selectively on HCT-116 cells, with selectivity index after 72 h: SI(**1d**) = 4.6; SI(**2d**)= 3.3; SI(**3d**) = 2.3. With the same modification, selectivity can be seen in group **5** after 24 h: SI(**5d**) = 2.0 with the recovery of both cell lines after 72 h. On the other hand, the compounds of series **5** slightly differ, where modification **c** shows the strongest cytotoxic effect on both cell lines, while modification **b** expresses the highest selectivity on cancer cells after both observed times in this group (table 9). These findings also point out the significance of R^5^-OH substitution of the ring B on both cytotoxicity and selectivity towards cancer cell lines. The analogues of series **5** consist of a gallic acid fragment, i.e. the A ring bears three -OH groups, which significantly influences cell viability. Khaledi *et al*. also reported a beneficial effect of gallic acid on cytotoxicity in cancer cells [75]. According to the obtained results, the selective sensitivity of HCT-116 cells towards the compounds from group **5** of **Phe*N*AH**s is not so notable, with some exceptions, which can be seen in table 9.

**Table 9.** Selectivity index. SI values of **5a–h** group of hydrazones and commercial cytostatic drugs after 24 and 72 h exposure.

SI	**5a**	**5b**	**5c**	**5d**	**5e**	**5f**	**5g**	**5h**	leucovorin	irinotecan
24 h	0.71	1.67	1.28	2.00	1.00	0.97	0.76	1.00	1.00	1.00
72 h	0.93	1.61	0.83	<0.92	0.70	0.92	1.02	0.86	<0.36	0.35

The **c, e, f, g** and **h** modifications possess an -OH group in the R^7^ position of the B ring, which combined with gallic acid fragment influence the cells almost nonselectively, i.e. have a similar influence on both cell lines. According to this, it is desirable that there are no substituents in the R^7^ position of the B-ring, this way favouring a selective effect on the cancer cell. By contrast, in this group, we can again see that the presence of R^5^*-*OH substitution on the B ring is desirable, as indicated by the selectivity index of substances **5b** and **5d**.

In compounds of all series with modifications **b**, **d**, **g**, and **h** there is a decrease of cell viability in the dose and time-dependent manner, i.e. by increasing the substance concentration cell viability decreased which is more evident after 72 h from treatment. The mechanism presumed to lead to the cytotoxic effect of these substances is apoptosis described by several authors [76,77], i.e. cell cycle arrest in the G2/M phase resulting in apoptosis [78]. In figures S109–S118 (electronic supplementary material), the viability of cells in most cases decreases more with higher concentration and after prolonged exposure of cells to these compounds. However, in several cases of hydrazones, a stronger effect occurs after 24 h of exposure, which indicates the acute influence of these compounds, after which the cells recover. The representative example is in group **5**, modification **d**. This may indicate the development of various mechanisms of resistance.

In addition to the effects obtained by *in vitro* testing of **Phe*N*AH**s, the results for commercially available cytostatic drugs are provided (table 7 and electronic supplementary material, figures S119 and S120). As positive controls, Leucovorin and Irinotecan were selected, as compounds used in colon cancer treatment and whose structures resemble the structures of the tested compounds. IC_50_ values show that Leucovorin has no effect on HCT-116 cells, while the effect of Irinotecan is moderate, but only 72 h after treatment. At the same time, these two drugs show higher impact on healthy MRC-5 cells, especially Irinotecan after 72 h. This result is not surprising, because cytostatic treatment is almost always accompanied by side effects on healthy tissue [79,80]. On the other hand, the effect of individual cytostatic in the treatment of colon cancer is not crucial, especially given that the ‘protocols’ are based primarily on the synergism of different cytostatic drugs. Thus, one of the standard protocols for colon cancer treatment is *Folfiri*, which is a combination of Irinotecan, Leucovorin, and 5-Fluorouracil that are administered at different doses at a specific pace in repetitive cycles. Comparison of the obtained IC_50_ values for **Phe*N*AH**s and commercial cytostatic drugs on cancer cell lines revealed that the IC_50_ values of Irinotecan and Leucovorin are generally similar to or higher than those for the **Phe*N*AH**s. This implies that smaller doses of **Phe*N*AH**s are needed to achieve the effect. On the other hand, the doses required to inhibit the growth of 50% of healthy cells (MRC-5) are generally higher in **Phe*N*AH**s than in commercial cytotoxic drugs. This is in favour of **Phe*N*AH** derivatives because it indicates that they are more selective for cancer cells compared to commercially available drugs (tables 7–9).

## 4. Conclusion

In the present work, the synthesis of five series of phenolic *N*-acylhydrazones (with a total of 40 **Phe*N*AH** compounds) was performed starting from the corresponding benzohydrazides and various aromatic aldehydes. The obtained products were characterized experimentally by NMR, IR and UV-Vis methods, and theoretically using density functional theory (DFT). The assessment of antioxidant properties of the **Phe*N*AH** derivatives revealed that 20 out of 40 synthesized analogues were active toward DPPH radical. All analogues of series **5** expressed excellent scavenging activity toward DPPH radical, with the IC_50_ value in the range of 0.7–5.9 µM. The best antioxidant capacity expressed analogue **5h** with IC_50_ = 0.7 µM. The calculated stoichiometric factor (SF) value in the range from 4.3 to 17.9 designated all analogues of series **5** as excellent antioxidants. On the other hand, derivatives **e**, **f**, and **h** of series **1–4** also exhibited significant radical scavenging ability, where the best results were obtained for catechol-type derivatives **h**. Furthermore, DFT investigations were performed to elucidate the antioxidant capability of all compounds from a thermodynamical aspect, as well as to get insight into the preferable antioxidant mechanism, both in the presence and absence of free radicals. Excellent agreement between experimental and theoretical data was achieved. Calculated electronic properties (energies of the HOMO and LUMO, as well stabilization energies Δ*E*_iso_) pointed out **e**, **f**, and **h** derivatives (vanillin-, syringaldehyde- and catechol-like analogues) of series **1–5** as ones with the most favourable thermodynamical parameters for radical scavenging. On the other hand, in the absence of free radicals, bond dissociation enthalpy (BDE), ionization potential (IP), proton affinity (PA), proton dissociation enthalpy (PDE) and electron transfer enthalpy (ETE) values indicated the SPLET mechanism as prevailing in polar solvents, whereas the HAT-SPLET competition was observed in nonpolar surroundings. In the presence of medically relevant radical species, the mutual comparison of the calculated Δ*H*_BDE_, Δ*H*_IP_ and Δ*H*_PDE_, Δ*H*_PA_ and Δ*H*_ETE_ values suggested mainly HAT-SPLET competition in water and methanol, while the SPLET pathway is mostly prevailing in benzene as solvent.

The **Phe*N*AH** derivatives were evaluated for their ability to inhibit the growth of HCT-116 and MRC-5 cell lines, also. Obtained results reveal the influence of the type and position of the functional groups on both cytotoxicity and selectivity towards cancer cells. All compounds bearing –OH group in the R^5^ position of the B ring expressed enhanced cytotoxic effects, as well as increased selectivity on cancer cells. Moreover, all compounds from series **5** induced a decrease in cell viability with almost no selectivity, except compounds **5b** and **5d**, where the B ring bears R^5^-OH substitution. The IC_50_ values for commercial cytostatic drugs Leucovorin and Irinotecan for HCT-116 cell line were similar or higher than those for the **Phe*N*AH**s. On the other hand, the doses required to inhibit the growth of 50% of MRC-5 cells are generally higher for **Phe*N*AH**s than for commercial cytotoxic drugs. This points out that smaller doses of **Phe*N*AH**s are needed to achieve the effect, as well as increased selectivity of **Phe*N*AH**s towards cancer cells. The present results highlighted synthesized hydrazone derivatives as an excellent base for the design of new anti-cancer agents.

## Data Availability

The datasets supporting this article have been uploaded as part of the electronic supplementary material [81].
